# Complete Genome Sequence Analysis and Characterization of Selected Iron Regulation Genes of *Pasteurella Multocida* Serotype A Strain *PMTB2.1*

**DOI:** 10.3390/genes10020081

**Published:** 2019-01-25

**Authors:** Shagufta Jabeen, Huan Y. Yap, Faez Firdaus J. Abdullah, Zunita Zakaria, Nurulfiza M. Isa, Yung C. Tan, Yap S. Joo, Dilan A. Satharasinghe, Abdul R. Omar

**Affiliations:** 1 Institute of Bioscience, Universiti Putra Malaysia, 43400 Serdang, Selangor, Malaysia; sjabeen88@yahoo.com (S.J.); icoolhy@gmail.com (H.Y.Y.); zunita@upm.edu.my (Z.Z.); nurulfiza@upm.edu.my (N.M.I.); dilansatharasinghe@yahoo.com (D.A.S.); 2 Faculty of Veterinary Medicine, Universiti Putra Malaysia, 43400 Serdang, Selangor, Malaysia; jesse@upm.edu.my; 3 Faculty of Biotechnology and Bimolecular Sciences, Universiti Putra Malaysia, 43400 Serdang, Selangor, Malaysia; 4 Codon Genomics S/B, Jalan Dutamas 7, 43200 Seri Kembangan, Selangor, Malaysia; yungchie@gmail.com (Y.C.T.); ivan.hoh@codongenomics.com (Y.S.J.)

**Keywords:** comparative genomics, DNA sequencing, phylogenomics, phage, iron-regulating genes, real-time PCR, gene expression profiling, fold changes

## Abstract

Although more than 100 genome sequences of *Pasteurella multocida* are available, comprehensive and complete genome sequence analysis is limited. This study describes the analysis of complete genome sequence and pathogenomics of *P. multocida* strain *PMTB2.1*. The genome of *PMTB2.1* has 2176 genes with more than 40 coding sequences associated with iron regulation and 140 virulence genes including the complete *tad* locus. The *tad* locus includes several previously uncharacterized genes such as *flp2*, *rcpC* and *tadV* genes. A transposable phage resembling to Mu phages was identified in *P. multocida* that has not been identified in any other serotype yet. The multi-locus sequence typing analysis assigned the *PMTB2.1* genome sequence as type ST101, while the comparative genome analysis showed that *PMTB2.1* is closely related to other *P. multocida* strains with the genomic distance of less than 0.13. The expression profiling of iron regulating-genes of *PMTB2.1* was characterized under iron-limited environment. Results showed significant changes in the expression profiles of iron-regulating genes (*p* < 0.05) whereas the highest expression of *fecE* gene (281 fold) at 30 min suggests utilization of the outer-membrane proteins system in iron acquisition at an early stage of growth. This study showed the phylogenomic relatedness of *P. multocida* and improved annotation of important genes and functional characterization of iron-regulating genes of importance to the bacterial growth.

## 1. Introduction

*Pasteurella multocida* (PM) species of the genus *Pasteurella* are Gram-negative facultative anaerobic bacteria belonging to the family *Pasteurellaceae* [1]. Based on its capsular antigen, the *P*. *multocida* is grouped into A, B, D, E and F capsular types and further classified into 16 serotypes (1–16) based on lipopolysaccharide (LPS) antigen [2,3]. *P. multocida* was earlier identified as a commensal in the upper respiratory tract of mammals and birds. However, *P. multocida* is often associated with acute as well as chronic infections in avian and bovine that can lead to significant morbidity and mortality [4,5]. Fowl cholera in avian, atrophic rhinitis in pigs and pasteurellosis characterized as respiratory diseases namely pneumonia and hemorrhagic septicemia (HS) in cattle and buffaloes are the most important veterinary diseases [6,7]. In general, pasteurellosis caused by *P. multocida* is an acute septicemic disease characterized by high morbidity and is a high-impact disease in livestock, according to the World Organization for Animal Health [7].

*Pasteurella multocida* serotype A:3 has been implicated in fatal pneumonia of cattle in India [8]. Furthermore, *P. multocida* serotype A:1 has also been reported to be responsible for respiratory diseases such as fatal pneumonia and septicemia of cattle and buffaloes [7,9]. Unlike pasteurellosis associated with pneumonia and septicemia induced by serotype A, HS is an acute septicemic disease caused by *P. multocida* serotypes B:2 and E:2 in cattle and buffaloes [6,7] that results in an outbreak with 100% mortality in infected animals [10]. Hence, HS and pneumonic septicemic pasteurellosis are considered to be the most important bacterial diseases economically in various countries in Asia and South Africa [10,11].

Currently, about 121 complete or draft *P. multocida* genomes are publicly available in the GenBank, National Center for Biotechnology Information (NCBI). However, only a few genomes have been studied and examined in detail such as *PM70* (serotype F) [12], *PM36950* (serotype A) [13], *PMHB01* (serotype A) [14] and the genomes of *P. multocida* strains harboring the *P. multocida* toxin (PMT) gene such as *PMHN06* (serotype D) [15]. The first complete genomic structure analysis of *P*. *multocida PM70* revealed that it possesses some predicted virulence genes and iron-uptake genes and a complete set of the genes for metabolic pathways necessary for the metabolism of certain carbohydrates and synthesis of all 20 amino acids [12]. Subsequently, the first integrative conjugative element (ICE) harboring antibiotic resistance genes were identified from the complete genome sequence analysis of *P*. *multocida PM36950* [13].

Iron is one of the most important nutrients for pathogenic bacteria like *Pasteurella* and the level of free iron available in the host is very limited [4,16,17]. It is possible that iron acquisition in *P. multocida* plays an important role in its survival and pathogenesis of the disease [4,16]. To this end, *P. multocida* strains have siderophore-independent iron acquisition systems homologous to the *Actinobacillus AfeABCD* system and the periplasmic binding protein-dependent iron transport systems homologous to *Escherichia coli FecBCDE* and *Neisseria FbpABC* systems [4]. The presence of multiple iron acquisition systems in the *Pasteurella* species may account for their ability to acquire iron under iron-limited conditions by utilizing a certain set of the genes under certain conditions. The gene expression patterns of *P. multocida* in iron-free chemically defined medium and in response to different iron sources have been studied by DNA microarray [17]. In another DNA microarray-based study [12], more than 150 genes showed more than two-fold altered expression when the *P. multocida* strain *PM70* was grown with an iron chelating agent. In both of the above studies, the transcriptional responses of *P. multocida* to either low iron or defined iron sources have been studied using microarray-based technology. However, the expression profiles of these genes were not characterized further based on the real-time PCR assay.

Recently, the *P*. *multocida* strain *HB01* complete genome sequence was produced using the next-generation sequencing (NGS) technology and primer walking Sanger sequencing [14], whereas, the *P*. *multocida* strain Razi 0001 (CP 017961.1) was produced as in silico complete genome sequence using the Pacific Biosciences (PacBio, Menlo Park, CA, USA) sequencing technology. The development of a third-generation sequencing technology such as PacBio, which is a long-read sequencing platform of relatively low cost and high throughput have greatly influenced the field of bacterial genome sequencing [18]. Recently we have isolated, *P. multocida* strain *PMTB2.1* based on the *Pasteurella multocida* specific PCR (PM-PCR) [19], from buffalo that died of septicemia. However, the genomic characteristics of the isolate are not known. Thus, the aim of this study was to perform a comprehensive genome sequence analysis of *PMTB2.1* and to study the gene expression pattern of selected iron-regulating genes namely *fecE*, *yfeA*, *fbpB* and *fur* as well as the colonization gene *nanA* (sialidase) to elucidate the actual response of these genes to the iron limiting condition.

## 2. Materials and Methods

### 2.1. Bacterial Strain and DNA Extraction

The *P. multocida* strain *PMTB2.1* was isolated from a buffalo that died of septicemia in Malaysia [20] and was identified by species-specific PCR [7,19]. The bacteria were prepared from a pure stock culture that was grown in brain heart infusion (BHI) broth at 37 °C for 12–16 h. The *PMTB2.1* genomic DNA was extracted using the DNeasy Mini spin column (Qiagen, Hilden, Germany). The extracted DNA was quantified and qualified using an Eppendorf Biospectrometer^®^ (Eppendorf, Hamburg, Germany) and agarose gel electrophoresis stained with 1× RedSafe^TM^ (iNTRon Biotechnology, Gyeonggi-do, Korea).

### 2.2. Genome Sequencing and Assembly

Genome sequencing was performed using the PacBio RS 2 sequencing platform (University of Malaya, Kuala Lumpur, Malaysia). The genomic DNA of *PMTB2.1* was sequenced using the C2 chemistry [21] and primary filtering was performed on the RS Blade Centre server following which secondary analysis was performed using the SMRT analysis pipeline version 1.4 (PacBio). The raw sequencing data were used to extract circular consensus sequence (CCS) and continuous long read (CLR). Error correction of the CLR reads, based on the CCS reads was carried out by the PacBio ToCA module of Whole-Genome Shotgun Assembler v8.2 with 26× coverage. These error-corrected reads were then assembled by the Celera Assembler. The genome was assembled de novo by runCA (Celera Assembler) (http://wgs-assembler.sourceforge.net) using default parameters, which generated three contigs. The contigs were then aligned together and assembled into a single contig with minimum gaps, based on the overlapping layout method. Primers were designed to sequence the gaps between the contigs. The Sanger sequencing method was utilized to sequence amplicons by primer walking strategy. The first and last nucleotide position of the circular genome of *PMTB2.1* was set based on homology to the reference genome of *P. multocida* strain *PM36950* (accession no. CP003022.1) and circularity chromosome was confirmed by PCR.

### 2.3. Genome Annotation and Genomic Organization of PMTB2.1

Genome annotation of *P. multocida* strain *PMTB2.1* was carried out using the Prokaryotic Genome Automatic Annotation Pipeline (PGAAP) provided by NCBI (http://ncbi.nlm.nih.gov). Genes were predicted using GeneMarks [22]; whereby the annotated sequences include CDs (coding sequences), ribosomal RNA (rRNA), transfer RNA (tRNA) and ncRNA (non-coding RNA). Functional annotations of the putative coding genes were predicted by PGAAP, NCBI using Basic Local Alignment Tools (BLAST) against non-redundant (nr) and Swiss-Prot (SP) protein databases [23] at a cut-off E-value ≤ 1 × 10^−5^ using Blast2GO [24]. Annotation was also carried out using the gene ontology Kyoto Encyclopedia of Genes and Genomes (KEGG) database [25]. Artemis [26] was used to visualize and analyze structural and functional annotation of the genome sequence of *PMTB2.1*. The origin of replication was determined using Ori-Finder [27], while the circular genomic plot of *PMTB2.1* was plotted by Circos version 0.69 [28] and the genomic islands were identified using online software IslandViewer 3 [29].

### 2.4. Genome Sequence Analysis

The analysis of complete genome sequence of *PMTB2.1* was performed to identify genes and sequence motifs of interest based on different databases. The potential virulence genes and antibiotic resistance genes were identified based on a homology search against Pathogen-Host Interaction Database (PHI-base) [30], Virulence Factors Database (VFDB) [31] and the Comprehensive Antibiotic Resistance Database (CARD) [32]. Phage and prophage-like sequences were predicted by PHAST [33]. Cut-off value of E-value ≤ 1 × 10^−5^ and subject coverage ≥ 70% and identity between two proteins ≥ 50% were applied to the blast p search. Multi-locus sequence typing (MLST) [34] was performed based on PubMLST (https://publst.org/multocida) and Centre of Genomic Epidemiology (CGE, https://cge.cbs.dtu.dk/services/MLST) against Rural Industries Research and Development Corporation (RIRDC) scheme.

### 2.5. Comparative Genomics of PMTB2.1

Comparative analysis was carried out on *PMTB2.1* and another eight selected genomes. The selected genomes were comprised of five complete genomes of *P. multocida* belonging to different serotypes; A, D and F from different host origins and the remaining three included *Actinobacillus pleuropneumoniae* JL03 (CP000687), *Haemophilus parasuis* SH0165 (CP001321) and *Escherichia coli* K12 MG1655 (NC_000913). Genome distance matrix was obtained by Progressive Mauve v2.3.1 [35] and visualized in a heatmap using R v3.0.2. Pairwise coverage values are subtracted from one to yield a distance value. The matrix is used to infer a guide tree and to scale the break-point penalty during anchoring. The synteny analysis was carried out using Mauve v2.3.1 [35] to compare complete genome sequences to identify regions of similarity and rearrangements.

Identification of the orthology groups of the genome was carried out using OrthoMCL v2.0.9 [36] and information obtained was used to construct a phylogenomic tree and to compare the selected genomes with *PMTB2.1*, using the Venn diagram software [37].

### 2.6. Expression Profiling of Iron-Regulating Genes of PMTB2.1

The *P. multocida* strain *PMTB2.1* cells were grown in 200 mL of fresh BHI broth with 2, 2’- Bipyridine (Merck, New Jersey, USA; 200 µM/mL). The cultures were incubated at 37 °C aerobically in a shaking incubator (Sartorius, Tagelswangen, Switzerland) and samples were removed from each cell culture at the time points of 30, 60, 120 and 180 min after incubation of the bacterial cell pellet. Bacterial cells were pelleted by centrifugation at 4000× rpm (Eppendorf Centrifuge 5415R, Hamburg, Germany) at 4 °C for 5–7 min, the supernatant was discarded and the resulted cell pellet was suspended in 2 mL of Bacterial RNAlater (Qiagen) and preserved at −80 °C for subsequent RNA extraction. The experiment was run in triplicate and bacterial cultures grown in BHI broth alone were maintained as controls.

Bacterial RNA extractions were performed with the RNeasy mini plus kit as recommended by the manufacturer (Qiagen). The quality of extracted bacterial RNA and their respective concentrations were checked using an Eppendorf Biospectrometer (Eppendorf, Germany). Reverse transcription PCR (RT-PCR) was carried out on a thermal cycler (Bio-Rad, California, USA) using the SensiFast cDNA synthesis kit (Bioline, London, UK) according to the manufacturer’s instructions. A total of four iron-regulating genes namely *fecE*, *yfeA*, *fbpB* and *fur* and a colonization gene *nanA* (sialidase) were selected to assess their relative mRNA expression using Taqman-based real time-PCR. Two housekeeping genes, glyceraldehyde-3-phosphate-dehydrogenase (*GAPDH*) and DNA gyrase B (*gyrB*) were used to normalize the reactions. The primers and probes were designed using the *PMTB2.1* complete genome sequence. The sequences of the primers and probes and their melting temperatures are shown in Table 1. Standard curves for the target and housekeeping genes were generated using extracted RNA converted to cDNA by Taqman Probe-based real-time PCR.

The mean quantitative cycle value (Cq) for a selected gene at a time point was determined from three biological replicates each with three technical replicates. The relative fold changes of these genes from the treated samples at selected time points of treatment were compared to the control samples. Relative fold changes were calculated by the 2^−ΔΔCt^ method. The data analysis, normalization with housekeeping genes and the quantification of fold changes were carried out as described by Bustin et al. [38] using Bio-Rad CFX manager software v3.1 (Bio-Rad). All quantitative data were expressed as a mean ± standard deviation. Statistical significance of relative fold change expressions was calculated by comparison between groups at *p* ≤ 0.05.

### 2.7. Data Availability

The complete genome sequence of *P. multocida* strain *PMTB2.1* and eight selected genomes for comparative analysis are available on NCBI and can be accessed using the accession number listed in the table with title “Comparison of *P. multocida* strain *PMTB2.1* and complete genome sequences of other *P. multocida* of different serotypes”, in the result section.

## 3. Results

### 3.1. Genome Assembly

A total of 49,844 CCS reads and 523,361 CLR were extracted from the PacBio raw sequencing data. Error correction of the CLR reads using CCS reads generated 13,231 reads with a mean read length of 2000 bp (total of 60,463,108 nucleotides) with 26× coverage. The genome was assembled de novo by runCA (Celera Assembler) using default parameters, which generated three contigs with the size of 1,057,336; 946,414; and 338,900 bp for each contig, respectively, to a total of 2,342,650 bp.

The unresolved gaps in the genome assembly were closed with the primer walking/Sanger sequencing reads by Staden v2.0.09. The circularity of the chromosome was confirmed by PCR where the trimmed sequences were successfully aligned with the complete genome sequence of *PMTB2.1* in such a way that the first 1,538 bp at the end of circle aligned with the last nucleotides at position 2,326,603 to 2,328,141. Meanwhile, the remaining 112 bp (in a circle) aligned at the nucleotide positions of 1 to 112 of the complete genome sequence of *P. multocida* strain *PMTB2.1*. The final finished grade, complete genome sequences of *P. multocida* strain *PMTB2.1* (2,315,138 bp) as a single contiguous circular chromosome was submitted to GenBank, NCBI under the accession number CP007205.2.

### 3.2. Genetic Organizations of the PMTB2.1 Complete Genome Sequence

**Figure 1:**
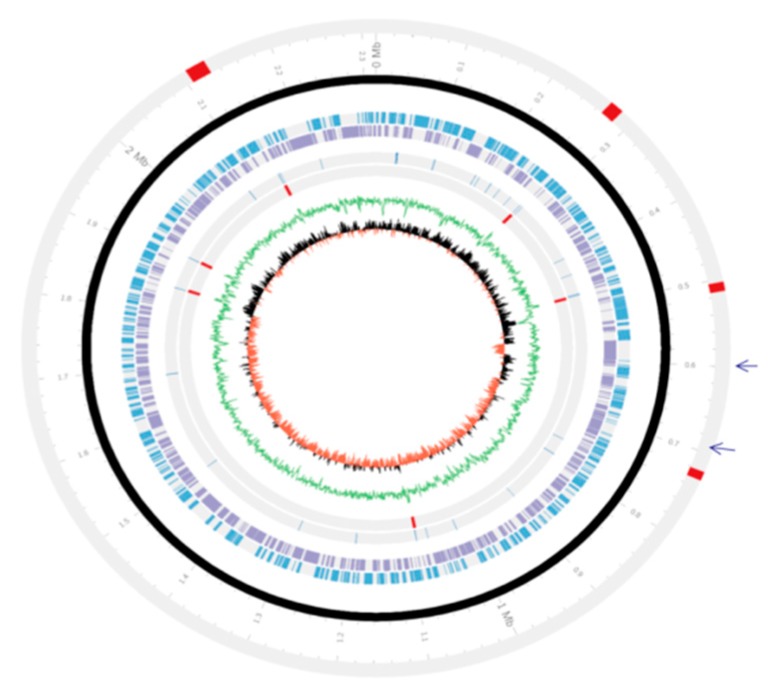
Circular map of the complete genome of *Pasteurella multocida* strain *PMTB2.1*. The arrangement of genes within the chromosome of *PMTB 2.1*. Genomic islands = Red block in outer grey line; Circular chromosome = Black circle; Forward genes = Sky blue circle; Reverse genes = Light violet circle; tRNA = Innermost greyline; rRNA = Red line on inner grey line; GC plot = Green circle; GC skew = Red half inner circle figure, two arrows indicate the position of phages.

The genome of *P*. *multocida* strain *PMTB2.1* is composed of 2,315,138 base pairs with a GC content of approximately 40.32% (Figure 1), containing 2097 protein-coding sequences, 19 rRNA (including 6, 16S rRNA operon), 56 tRNA and 4 ncRNA (non-coding RNA) genes, making a total of 2176 genes (Table 2).

The *dnaA* gene, designated AW43_0005 located between 101–1,456 nucleotides (nt), was selected as the first gene of the *PMTB2.1 genome* based on annotation of results of PGAAP. The putative replication origin (*oriC*) of the chromosome was identified by GC skew and DnaA-boxes immediately before the first putative coding sequence, *gidA*. The position of *oriC* is between 1,837,028–1,837,442 nt with 415 nucleotide length and closely situated to a first putative coding sequence *gidA* gene (1,837,443–1,839,332 nt).

### 3.3. Comparative Genomic Analysis and Genotyping of PMTB2.1

The *PMTB2.1* genome was compared against five complete genomes of *P. multocida* based on different serotype and host origin and three different genera of the family *Pasteurellaceae; Pasteurella* sp., *Actinobacillus* sp. and *Haemophilus* sp.; while *Escherichia* sp. represents the family *Enterobacteriaceae*. Genomic distance analysis showed that *PMTB2.1* is closely related to four other *Pasteurella* spp. with the distance of less than 0.12. The distance matrix of *PMTB2.1* to *PM70* is smaller (0.08) compared to that of *PMTB2.1* to *PM36950* (0.10, Table 3).

**Figure 2:**
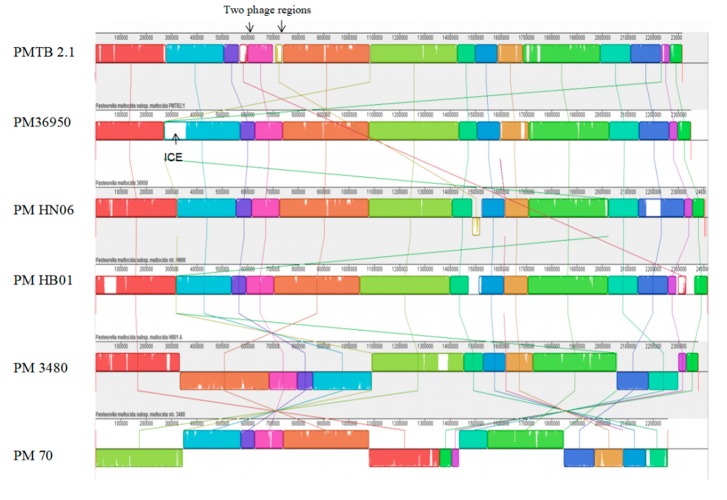
Synteny analysis of *P. multocida* strain *PMTB2.1* with other *Pasteurella* *multocida* complete genome sequences. ICE = integrative conjugative element.

In contrast, synteny analysis of different *P*. *multocida* genomes showed the genome structure of *PMTB2.1* resembles that of *PM36950* and reveals large structural variations between *PM3480* and *PM70* (Figure 2).

The similar color box represents the similar genetic structure, white gaps representing area specific to each isolate/genome whereas cross lines represent the reorientation of similar genetic structure differently among the different *P. multocida* genomes.

Furthermore, a genomic comparison of six selected *P*. *multocida* complete sequences including *PMTB2.1* is shown in Table 4, which revealed that *P*. *multocida* genomic size ranges between 2 and 2.4 Mbp in length, with a G+C content of between 40% to 41%. Moreover, the *PMTB2.1* complete genome sequence comparison with *P. multocida* reference strains *PM36950* and *PM70* shows that the *PMTB2.1* (2,315,138 bps) complete genome is approximately 34,380 kb smaller than *PM36950* (2,349,518 bps) and 57,651 kb larger than *PM70* (Table 4).

**Figure 3:**
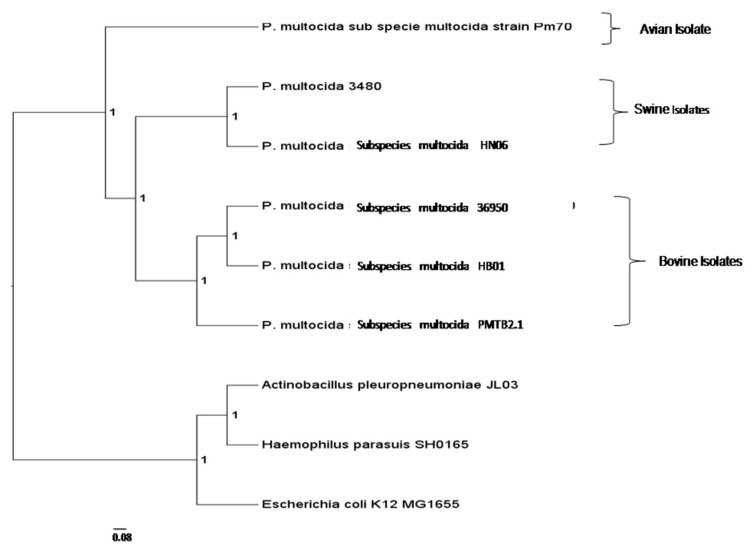
Phylogenetic analysis of *PMTB2.1* based on complete genome sequences. *PMTB2.1* was clustered closely with other *P. multocida* strain *36950* and *HB01* which were isolated from bovine.

A total of 20,630 protein sequences were retrieved from all nine genomes via the OrthoMCL analysis included in this study and clustered into 2831 orthologous clusters and 1025 single copy orthologous gene, which is used to determine the relatedness among *Pasteurella* strains from different host origins. The phylogenomic tree was constructed and rooted with *E. coli, A*. *pleuropneumoniae* and *H*. *parasuis*. The genome of *PMTB2.1* was clustered with that of *PM36950* and *PMHB01*, where both isolates are affecting bovine. They were separated from other *P*. *multocida* strains that infect avian and swine (Figure 3).

**Figure 4:**
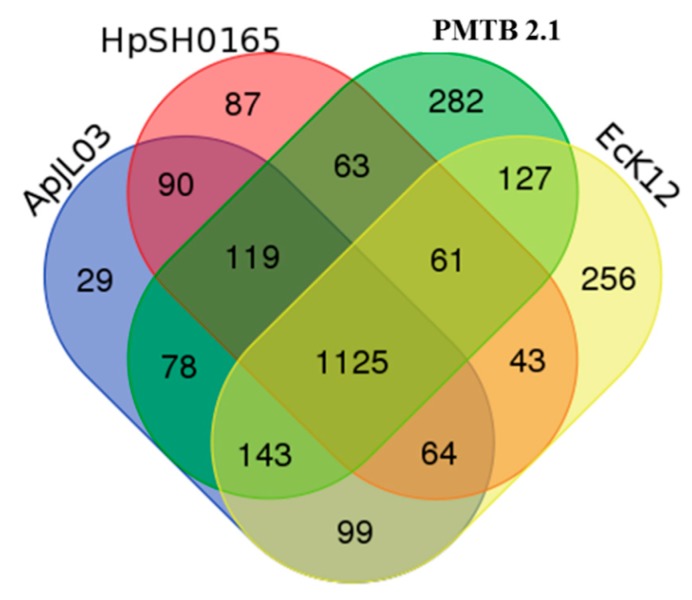
Shared genes and strain-specific genes of *PMTB2.1* compared to other selected genomes. strain-specific and shared genes among *Pasteurella multocida* strain *PMTB2.1* and *Haemophilus* (SH0165), *Escherichia coli* (EcK12) and *Actinobacillus pleuropneumoniae* (APJL03).

The analysis of the *PMTB2.1* sequence comparison based on the OrthoMCL analysis identified 282 CDs specific to *P*. *multocida* and 1,125 CDs with orthologs in all the *Haemophilus SH0165 (Hi SH0165*), *E. coli* (*Ec.K*12) and *A. pleuropneumoniae JL03* (*ActJL03*) genomes (Figure 4).

**Figure 5:**
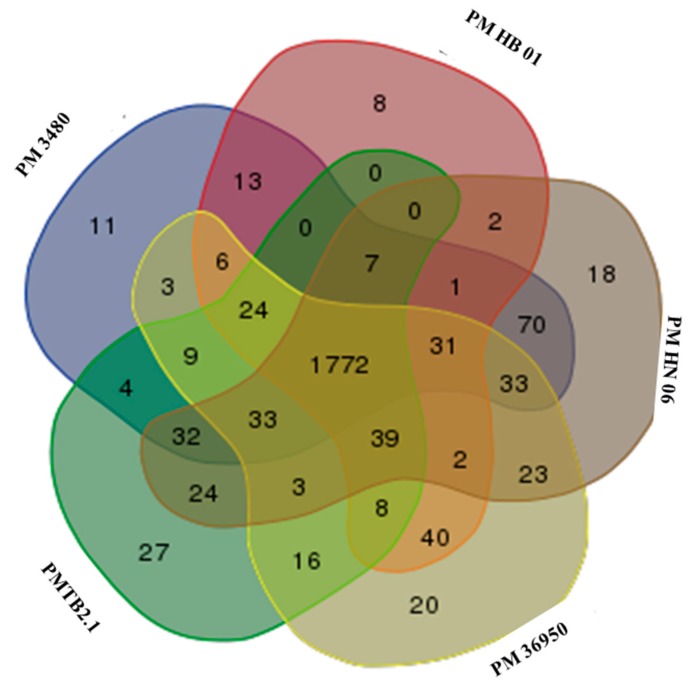
Genomic comparison based on ortholog group of the genes among *P. multocida*. Venn diagram showing the number of shared and strain-specific genes of each of the analyzed *Pasteurella* genome with 1772 common genes; *P. multocida* strain *PMHN06* and *PM3480* have 18 and 11 strain-specific genes, respectively. *PMTB 2.1* has more strain- specific genes, 27, as compared to *PM36950* and *PMHB01* which have 20 and 8 strain-specific genes, respectively.

Meanwhile, the genome comparison of *PMTB2.1* with other *Pasteurella* indicated 1772 genes were common to serotypes A and D with each species showing their own strain-specific genes (Figure 5). Moreover, multi-locus sequence typing (MLST) against RIRDC scheme showed *PMTB2.1* exactly matched the alleles of sequence type ST101 with 100% sequence similarity.

### 3.4. Virulence Genes and Antibiotic Resistance Genes of PMTB2.1

Potential virulence genes and antibiotic resistance genes were identified based on a homology search against the Pathogen-Host Interaction Database (PHI-base), which identified 32 virulence-associated genes and the Virulence Factor Database (VFDB), which identified 128 genes (Supplementary Figure S1) in the *PMTB2.1* genome. Among the virulence genes identified of particular importance are those which are involved in colonization such as the *tad* locus and capsular and LPS antigen (Supplementary Table S1). The presence of the *tad* locus gene in *PMTB2.1* was identified from the VFDB analysis results and genome annotation at the positions of 1,010,863 to 1,011,243 (gene AW43_04840 to gene AW43_04895), which included all of the 14 genes of *tad* locus genes [39] (Table 5).

The capsular (cap) locus in *PMTB2.1*, responsible for capsule biosynthesis and export is an approximately 14.9-kb region that encodes ten genes, genes AW43_04445 to gene AW43_04490 (911,463 to 926,403). In addition, the entire capsular sequence of the *PMTB2.1*, has 99% similarity to *P*. *multocida* serogroup A1 (accession no. AF067175.2). We have also identified the complete set of genes encoding enzymes for LPS biosynthesis in the genome of *PMTB2.1* based on the KEGG pathway analysis. Included in these genes are those genes involved in lipid A biosynthetic process and were identified in *PMTB2.1* from the VFDB analysis (Supplementary Table S1).

Furthermore, the Comprehensive Antibiotic Resistance Database (CARD) identified 12 genes (Supplementary Table S2) in *PMTB2.1* that can bring about resistance against a specific group of antibiotics. Among these antibiotic resistance genes, genes AW43_04995 and AW43_08130 were similar to tetracycline resistance genes *tet*(35) and *tet*(34), respectively, whilst gene AW 43_04465 was similar to *PmrE* of polymyxin resistance operon (Pmr). However, the sensitivity tests against tetracycline and ampicillin (to which most *Pasturella multocida* strains are fully sensitive) using disk diffusion method found that *PMTB2.1* was resistant to tetracycline and sensitive to ampicillin (data not shown) based on the zone size measured according to the British Society for Antimicrobial Chemotherapy (BSAC) guidelines [40].

### 3.5. Phage and Plasmid Sequences of PMTB2.1

Two apparently intact prophage sequences of approximately 62 kb designated as region 1 at positions 567,179–599,404 and region 2 at position 700,934–730,776 were detected in the *PMTB2.1* genome (Figure 1). One of the phages (region 1) is similar to transposable Mu-like phage SfMu; however, the phage regions of *PMTB2.1* were not associated with toxin-related genes, as detected in serotype D toxigenic strain of *P. multocida*. In addition, no plasmid sequence was detected in the complete genome sequence of *PMTB2.1*.

### 3.6. Iron Regulating Genes and Expression Profiling of Selected Iron-Regulating Genes in PMTB2.1

A total of five *P. multocida* strain *PMTB2.1* genes were assessed for their expressions under iron-limited environment using TaqMan-based real-time PCR (qRT-PCR) analysis. The efficiency of the PCR and the co-efficient of the variance (R^2^) of the real-time PCR assays were between 90%–105% and ≥ 0.98, respectively (Supplementary Table S3). *PMTB2.1* grown in iron-limited environment undergo significant changes (*p* < 0.05) in the expression profile of all of the four selected iron-regulating genes namely *fecE*, *yfeA*, *fbpB* and *fur* when compared to control (normal broth). Results of this study reflect that iron-limited environment has a significant effect on the expression profile of iron regulating-genes (*p* < 0.05) and all evaluated genes act differently in response to an iron limitation in the media (Table 6).

The *fur* gene, a universal transcriptional regulator of iron-regulating genes, showed a significant increase in the relative fold changes expression in the iron-limited environment at all time points (2–5 fold) and started to be expressed early at 30 min of treatment (Table 6). The iron-regulating gene *fecE* (AW43_06150) of the FecABCDE periplasmic binding protein-dependent iron transport systems, was highly expressed (281 fold) after 30 min of treatment, which started to decrease remarkably, from 4.7 to −1.5-fold at 60 and 120 min, respectively, with elevated expression of other iron-regulating genes namely *fbpB* (AW43_01110) and *yfeA* (AW43_01910) of the *FbpABC* and *yfeABCD* system which are outer-membrane independent ABC iron transport system. The *fbpB* gene was significantly expressed at a higher level of relative fold change (25 fold) at 60 min (Table 6), whereas the *yfeA* gene was significantly expressed with the highest relative fold change expression (42 fold) at 120 min (Table 6). On the other hand, the relative fold change expression of *nanA* (sialidase) in the treated samples collected at all time points were low, that is, less than 2-fold (Table 6) that showed the treatment has no significant effect on the *nanA* gene expression. 

## 4. Discussion

### 4.1. Complete Genome Sequencing

Long-read sequencing technologies such as PacBio RS can resolve repeat sequences that are found in most microbial genomes [21]. Hence, in this study, de novo assembly of the *PMTB2.1* generated a near-complete genome with gaps that were closed by Sanger sequencing and a complete circular genome (2,315,138 bps) was produced based on the reference genome sequence of *P. multocida* strain *PM36950.* The genomic size of single circular genome sequences of *P*. *multocida* belonging to different serotypes available on NCBI, ranges from 2 and 2.4 Mbp with a G+C content of 40%–41% [16], which is also reflected in the genomic comparison of six selected *P*. *multocida* complete sequences including *PMTB2.1* (Table 4).

### 4.2. Pathogenomics Analysis

**Figure 6:**
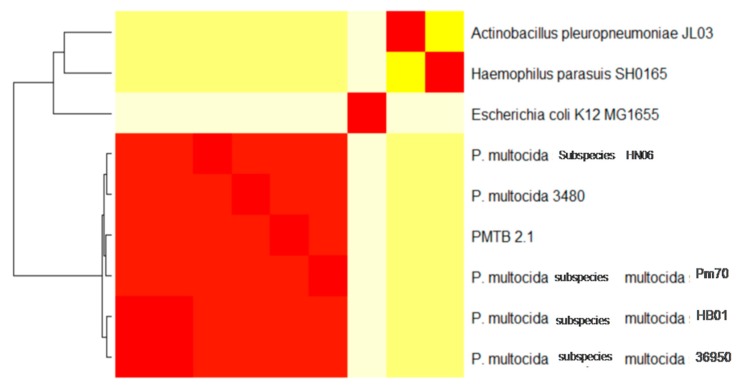
Genomic distance based on heat map analysis of *PMTB2.1* compared to other nine selected genomes. PMTB2.1 is grouped together with other *P. multocida* with genomic distance less than 0.13 (red color box), while *Haemophilus* and *Actinobacillus* are groups together with similar genomic distance 0.72 (lemon yellow color box) and *E. coli* with genomic distance 0.80 (off-white color box) Red color box in off-white *E. coli* bar indicating genomic region similar to *Pasteurella*, same for *Haemophilus* and *Actinobacillus*.

Synteny analysis based on similarity of the genetic structure of different *P*. *multocida* genomes (Figure 2) revealed subtle differences in genetic structures indicating the dynamics of frequent gene transfer events among the pool of *P*. *multocida* strains. However, *PM3480* and *PM70* exhibited exceptionally large structural variations, as *PM70* is an avian isolate (isolated from chicken) and *PM3480* is a swine isolate, whereas *PM36950*, *HB01* and *PMTB2.1* are of bovine origin. However, the genomic contents of *PMTB2.1* resemble more of the *PM70* strain with a distance matrix of 0.08, while the genomic structure resembles more of the *PM36950* strain with a distance matrix of 0.10, as compared to other *Pasteurella* strain (Table 3 and Figure 2). Furthermore, genomic distance analysis showed that *PMTB2.1* is closely related to the other four *Pasteurella* spp. causing it to be grouped in the heatmap analysis with the distance of less than 0.12 (Figure 6).

The genome comparison of *PMTB2.1* with five *Pasteurella* strains identified 1772 common genes among serotypes A and D (Figure 5). However, strain-specific genes belonging to each serotype and strains were present in all *P. multocida* genomes included in the analysis. *P. multocida* strain *PMHN06* and *PM3480* were found to have 18 and 11 strain-specific genes, respectively. As each strain has their own strain-specific genes, *PMTB 2.1* has more strain-specific genes (27 genes) compared to another bovine serotype A, *PM36950* with 8 and *PMHB01* with 20 strain-specific genes; this probably reflects strain specificity of different *Pasteurella* even from the same serogroup of the similar host (bovine).

**Figure 7:**
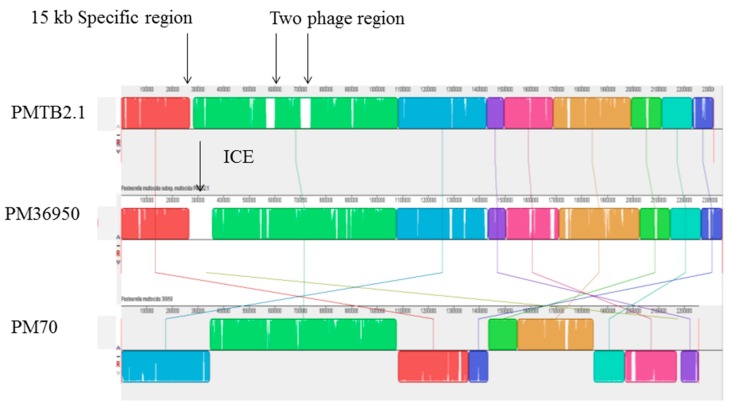
Synteny analysis (co-linearity comparison) between *PMTB2.1*, *PM70* and *PM36950*. Blocks with similar color represent similar sequences, while block with similar colors but with different orientation as in *PM70* exhibit structural variation. *PMTB2.1* specific two phage region and ICE region of *PM36950* is represented by white color or gaps between nucleotide blocks indicated with arrows.

Strain-specific integrative conjugative element (ICE), ICE*Pmu1* of 82 kbp found to be present only in *PM36950* [13], was absent in *PMTB2.1* and *PM70* (Figure 7). Meanwhile, 62,069 kb region (two prophage regions) was found to be present only in *PMTB2.1* but was absent in *PM36950*. Moreover, although the *PMTB2.1* genetic content more resembled *PM70* compared to other PM strains, *PMTB2.1* was found to have an extra 15-kb region (AW43_01320–AW43_01390), which was not present in the *PM70* genome (Figure 7).

Remarkably, the number of orthologous genes/proteins shared between the bovine strains *PMTB2.1* and the avian strain *PM70* (65 genes) was also greater than those shared between the two bovine isolates *PMTB2.1* and *PM36950* (only 22 genes). In addition, the numbers of virulence-associated genes identified in the complete genome sequence of *PMTB2.1* are 128, 32 and 12 based on the VFDB, PHI-base and CARD databases, respectively, are higher compared to previous studies [12]. One of the possible reasons is due to the high quality of genome sequences produced in this study.

Genome sequence comparison of *P. multocida* strain *PMTB2.1* with *Haemophilus SH0165*, *E. coli* (*Ec.K*12) and *A. pleuropneumoniae JL03* genomes identified 1125 ortholog CDs (Figure 4), reflecting overall close relationship and support the classification of these bacteria in the gamma subdivision of the *Proteobacteria*. Interestingly, the phylogenetic relationship within the *Pasteurellaceae* family based on 16S rRNA genes suggested that the *Pasteurella* species isolates are more closely related to the isolates of the genus *Haemophilus* [4], however the ortholog gene analysis shows that *P. multocida PMTB2.1* shared a greater number of ortholog proteins with *A. pleuropneumoniae* JL03 (78 genes) compared to *H. parasuis SH0165* (63 genes, Figure 4).

### 4.3. Phylogenomics Analysis

A total of 1025 single copy orthologous genes were used to determine the relatedness among different *Pasteurella* strains from different host origin that included *PM70* avian isolate [12], *PM3480* (unpublished) and *HN06* [15] swine isolates and *PM36950* [13], *HB01* [14] and *PMTB2.1* bovine isolates. The phylogenomic tree was constructed and rooted with *E. coli*, *A. pleuropneumoniae* and *H. parasuis*. The genome of *PMTB2.1* was clustered with that of *P. multocida* strain *PM36950* and *HB01*, where both isolates are of bovine origin (Figure 3). They were separated from other *P. multocida* strains that infect avian (*PM70*) and swine (*P. multocida* strain *3480* and *HN06*) and are distant from *Actinobacillus* and *Haemophilus*.

Hence, based on the complete genome sequences, a clear phylogenomic relatedness of *P. multocida* and its host can be established that was not possible in previous studies based on single nucleotide polymorphisms (SNPs) analysis [16] or MLST analysis [34]. In addition, a recent study based on genome-wide SNPs, at positions that were conserved across different *P. multocida* strains namely 17 HS-associated strains and 5-fowl cholera isolates including *PM70* and X73, the swine isolates *HN06* and *3480* and the bovine respiratory disease isolate *PM36950*, is still unable to show a clear correlation between strain relatedness and disease type other than for the HS strains [41]. This reflects that the complete genome sequence analysis proved useful to establish phylogenomic relatedness among the complete genomes of *P. multocida*.

### 4.4. Antibiotic Resistance Genes

*Pasteurella multocida* is an ampicillin-sensitive Gram-negative bacterium. Antimicrobial susceptibility study of *P. multocida* (108 avian isolates) by agar disc diffusion method showed that more than 90% of these isolates exhibited sensitivity to ampicillin [42]. However, it has been documented that *P. multocida* has developed resistance to commonly used antimicrobial agents. The antimicrobial sensitivity pattern of 56 *P. multocida* strains isolated from poultry reported exceptional resistance to sulphonamides, tetracyclines, first-generation quinolones and aminoglycosides [43]. The presence of *tet*(*H*) gene on the chromosome that belongs to the efflux-mediated class of gene for tetracycline resistance was detected in an avian strain of *P. multocida* [44] and later in other strains of *P. multocida* and *P. hemolytic* [45].

*Pasteurella multocida* strain *PMTB2.1* possess genes *tet*(*34*) and gene *tet*(*35*) that are predicted to encode resistance to tetracycline. Tetracycline resistance is most often due to genes, which codes for the efflux of tetracycline or for a protein that protects the bacterial ribosome from the action of tetracycline. The genes *tet*(35) and *tet*(34) are mostly found in Gram-negative bacteria such as *Vibrio*, *Pseudomonas*, *Serratia* and *Stenotrophomonas* [46]. Moreover, the integrative conjugative element (ICE*Pmu1*) of *PM36950* also contains genes conferring resistance to tetracycline. Along with the gene responsible for tetracycline resistance, another gene AW43_04465 similar to PmrE has been identified in *PMTB2.1*. The protein product of *PmrE* gene is required for the synthesis of 4-amino-4-deoxy-l-arabinose (Ara4N) of lipid A, in Gram-negative bacteria. This, in turn, allows the bacterial cell to resist the antimicrobial activity of cationic antimicrobial peptides and antibiotic such as polymyxin. Resistance to polymyxin and cationic antimicrobial peptides in *E. coli* and *Salmonella typhimurium* is acquired by similar phenomenon [47].

### 4.5. Tad Locus Genes of PMTB2.1

The *tad* locus is generally a 14-gene locus (*flp1-flp2-tadV-rcpCABtadZABCDEFG*) (Table 5). The *Tad* locus cluster is widely present in many bacterial and archaeal species, encoding for a type IVb pilus and represents a subtype of type II secretion. The *tad* genes encode the machinery that is required for the assembly of adhesive Flp (fimbrial low-molecular-weight protein) pili [40]. *Tad* locus genes in *PMTB2.1* are similar (51%–87% amino acid identity) to *Aggregatibacter actinomycetemcomitans* strain CU1000 *tad* locus gene [48]. Complete genome analysis of *P. multocida* strain *PMTB2.1* was able to detect the complete *tad* loci in *P. multocida* genome with improved annotation (Supplementary Figure S3, Table 5) where genes such as *flp2* was annotated as a hypothetical protein in previous studies but was annotated as pilus assembly gene in *PMTB2.1.* In addition, comparison of *tad* locus among different strains of *P*. *multocida* [14] found that the *tad* locus in HB01, *PM70* and *PM36950* is composed of only 13 genes and missing the *flp2*. The *tad* locus of strain *36950* is more truncated than other *P*. *multocida* due to the lack of *rcpC* gene. However, the *tad* locus in *PMTB2.1* comprises all 14 genes including the *flp2* and *rcpC* genes.

*Tad* locus is essential for biofilm formation, colonization and pathogenesis in many members of the family *Pasteurellaceae* including the genera *Actinobacillus*, *Haemophilus* and *Pasteurella* [49]. The function and features of the products of several *tad* locus genes have been reviewed in various bacteria and it has been reported that at least 12 and probably 13 (excluding *flp2*), of the *tad* genes, are required for all adherence-related phenotypes [39], whereas *rcpB* and *tadV* are essential in adherence [48,49]. Studies have also revealed the *flp1* encodes the Flp1 prepilin [50] and *tadV* encodes a protease that processes the Flp1 prepilin to the mature Flp1 pilin for assembly of pili. The product of *rcpA* has the properties of an outer membrane pore (secretin) and the product of *rcpB* is an outer-membrane protein that may gate the pore [48]. The *tadZ* encodes a protein that may localize the *tad* secretion machine to a pole, whereas the *tadA* product is an ATPase [51] and the *tadE* and *tadF* products are “pseudopilins,” whose functions are not known; but the pseudopilins are processed by *tadV* in the same way as the prepilin [49].

The presence of a homologous *tad* locus in the genomes of all five selected *Pasteurella* strains (*PM70*, *3480*, *HN06*, *36950* and *HB01*) along with *PMTB2.1*, suggested that type IVb pili may play a role as a host colonization factor in the respiratory tract of ruminants and birds. Moreover, the presence of *tad* gene in *P*. *multocida* supports the idea of a pili-mediated host colonization and persistence mechanism for *Pasteurella*. In addition, tenacious adherence may be a property of *Pasteurella* because it must be able to colonize in the presence of extensive normal flora. Therefore, the strong phenotype of tenacious adherence may be a special property of the *tad* locus of *Pasteurella*. The analysis of *tad* loci in *P*. *multocida* genome is critically important to determine and understand the functions of the individual *tad* genes and whether and how the various proteins encoded by a *tad* locus acted to colonize a specific niche and the importance of the *tad* genes for *P*. *multocida* and other prokaryotes.

### 4.6. The Capsular and LPS Genes of PMTB2.1

The pathogenicity of *P. multocida* is associated with various virulence factors such as the capsule and the LPS [16]. NCBI blast revealed that the capsular region sequences of *PMTB2.1* are similar to serotype A capsular region [52] with 99% identity. In addition, the *cap* locus operon comprises four genes, (AW43_04445 to AW43_04460) is associated with capsule biosynthesis and is similar to *hya*EDCB, an operon in *P*. *multocida* serotype A:1. Downstream of *hyaEDCB* is another operon of four genes *hex*DCBA (AW43_04475 to AW43_04490), which form a complex involved in the export of capsule to the surface and three of these genes have a high level of similarity to *hex*DCA of *Haemophilus influenzae* strain *1007* while one gene is highly similar to *Ctrc* gene of *Neisseria meningitidis* strain *MC58*. Similar results have been obtained from a study carried out on bovine isolate *HB01*, a capsular serotype A strain, which has identified 10 genes within a region of approximately 14.9 kb [14].

*Pasteurella multocida* LPS plays a critical role in disease immunopathogenesis [53,54]. The typical structure of LPS produced by most *P*. *multocida* is primarily composed of lipid A, a highly hydrophobic lipid moiety and immunoreactive oligosaccharide that triggers defense-related responses and causes Gram-negative sepsis [14]. The biosynthesis of Kdo2-lipid A, the LPS substructure, involves nine enzymatic steps. However, not all Gram-negative bacteria have all nine enzymes. Previous studies have shown that most of the genes encodes for lipid A such as *lpxA*, *lpxB*, *lpxC*, *lpxD*, *lpxH*, *lpxK*, *kdsA kdsB kdtA*, *lpxM*, *kdsC* and *htrB* are conserved among *Actinobacillus* and *Haemophilus* [54] and are also highly similar among *Pasteurella* species [14]. The genome of *PMTB2.1* based on VFDB analysis encode for *lpxA, lpxB*, *lpxC*, *lpxD*, *lpxK*, *kdsA*, *kdsA*, *htrB*, *kdtA and kdkA*; whereas recently sequenced genome of *P. multocida* strain *HB01* was reported to have two genes namely *kdtA* and *kdkA* [14]. On the other hand, it has been reported that some Gram-negative bacteria have genes only for the first four enzymes *lpxA, lpxC, lpxD* and *lpxB* [55].

### 4.7. Bacteriophages in Pasteurella and Prophage Sequence in PMTB 2.1

Phage-related sequences have been reported from *P. multocida* serotype A strain, such as a temperate transducing phage F108 [56] and porcine toxigenic serotype D strain [15,57]. However, the only phage isolated from toxigenic strains were found to carry the gene for PMT toxin, a major virulence factor of atrophic rhinitis in pigs, which belongs to the *Siphoviridae* family of bacteriophage that includes lambda-like phage [57]. Recently, three regions of prophage sequences were detected in a bovine virulent isolate of *P. multocida* strain *HB01* by complete genome sequencing [14]. In this study, a transposable temperate Mu-like prophage sequence similar (67%) to SfMu-like bacteriophage belonging to the family *Myoviridae* [58] was identified in *Pasteurella* strain *PMTB 2.1.* Two regions designated as region 1 and region 2 with prophage sequences were detected in *PMTB2.1* using PHAST database (Supplementary Figure S4). Region 1 was predicted to be similar to SfMu accession no. NC_027382, which was isolated from *Shigella flexneri* [58], while region 2 did not have any significant similarity with the previously determined complete phage genome sequence.

### 4.8. Iron Regulating Genes and Expression Profiling of Selected Iron-Regulating Genes in PMTB2.1

Iron acts as a co-factor or prosthetic group in several essential enzymes [59] and is needed for amino acid, pyrimidine, DNA biosynthesis and participates in electron transport [60]. Results of this study reflect that iron-limited environment has a significant effect on the expression profile of iron-regulating genes (*p* < 0.05) and all evaluated genes act differently in response to an iron limitation in the media. The highest expression of *fecE* gene (281 fold) at an early stage of treatment (30 min, Table 6) suggests that in the initial stage of growth, *Pasteurella* utilizes the iron acquiring genes, which facilitate the direct import of ferrous iron to scavenge iron from the environment under iron-limited conditions. Utilization of the outer-membrane proteins system in iron acquisition at an early stage of infection may play an important role in the development of a vaccine against infection with *P*. *multocida* since many outer-membrane proteins involved in iron regulation are strong antigens and some of them have been used in protective experiments against bacterial pathogens [61].

Furthermore, the downregulation of relative expression of *fecE* gene in association with the increase in relative expression of *fbpB* and *yfeA* genes (Table 6) indicates that *Pasteurella* controls their iron requirements in response to iron availability by downregulating the expression of iron proteins during iron-restricted growth. This idea is supported by the high relative fold change expression of *fur* at 180 min (5.4 fold), when *PMTB2.1* has started to decrease the expressions of *fecE* and *fbpB*, with only one gene *yfeA* being upregulated. The yfeABCD and FbpABC encodes an ABC transport system, whose expression is iron and Fur regulated. The *fur* gene has shown significantly higher relative fold changes expression at all-time points (2‒5 fold) (Table 6) and start to express early at 30 min of treatment (4 fold).These results show that like in other Gram-negative bacterial cells such as *E. coli*, the *fur* gene in *P*. *multocida* is significantly involved in the regulation of iron-regulating genes and controls the intracellular concentration of iron, which is toxic to the bacterial cell in high concentration [62]. Almost similar results were reported by May et al. [12] when they examined the pattern of gene expression of known iron-regulating genes of *P*. *multocida* strain *PM70* under iron-depleted condition with microarray based study, with an overall increase in expression of *yfeA* and *fecE* genes at all time points tested (13).

Moreover, significantly higher relative fold change (25 fold) of the *fbpB* gene (ortholog of *fbpB* of *FbpABC* system) at 60 min is probably associated with the ability of *P*. *multocida* to directly use host iron complexes such as heme, hemoglobin, transferrin and lactoferrin. This suggestion is based on the previous study that showed that the *FbpABC* system in *Neisseria spp.* is involved in transporting iron, delivered by transferrin and lactoferrin across the cell membrane without the help of periplasmic protein and siderophore [63]. Besides these genes, the increase in relative expression (26 fold) of periplasmic chelated iron-binding protein gene, *yfeA* at an early time point (30 min) and highest relative expression (42 fold) at 120 min reflect the use of multiple iron acquiring systems in *P*. *multocida* as *yfeA* gene of *PMTB2.1* is similar to one of the *Yersinia pestis YfeABC* iron transport system gene *yfeA,* [64].

The expression of *nanA* (sialidase) gene of *PMTB2.1* was also assessed under iron-limited environment as this gene is involved in colonization. A previous study has shown that *nanA* encodes for sialidases that exhibits glycolytic activity on mucin and release terminal sialic acid residues that can then be used as a bacterial carbon source [65]. Compared to iron-regulating genes, the relative expression of *nanA* was low (Table 6), which shows that probably iron scarcity does not exhibit a significant effect on the colonization of *P. multocida* facilitated by the *nanA* gene. Although *P. multocida* strains producing sialidase have been reported, their role in the virulence of *P. multocida* has yet to be established [59,65].

## 5. Conclusions

Improved complete genome sequence analysis of *P*. *multocida* strain *PMTB2.1* has provided valuable insights on the genomic structure with detection and identification of key important genes namely antibiotic resistance genes, a novel transposable Mu phage and various virulence genes including the Tad locus. In addition, clear phylogenomic relatedness for *P*. *multocida* can be established based on the complete genome analysis. Findings from this study has open up further studies on elucidating the mechanisms behind the molecular pathogenesis of *P. multocida* associated diseases in animals.

## Figures and Tables

**Table 1:** *PMTB2.1* genes primer and probe sequences for real-time PCR assay.

Primer & Probe	Sequence (5’–3’)	Product Size (bp)	Annealing Temperature (°C)
*fbpB* F	GCTGCCATTGCAGGCTTAGG	209	61
*fbpB* R	GGTTGCCACGCGTGTATCTG		
*fbpB* probe	CGCTCAATGACCACGGTCAGCGCA		
*fecE* F	GGCTGCGGAAAATCCACGTT	152	49
*fecE* R	TGGTACCAGGTGTTGTTGTGGT		
*fecE* probe	GCGCTTGCACGTTTGCTGAAACCCA		
*fur* F	AGCGGGGTTGAAAATCACCG	267	49
*fur* R	CACTTTACCGCAGTCCACGC		
*fur* probe	ACTTGCCCCAACTGAACACCACGA		
*yfeA* F	ATTACCAAACCGGGTGCGGA	426	61
*yfeA* R	AGGCGCCTTCACTGGTTACT		
*yfeA* probe	ACCGGCTGTCGTTGTGACAGAAGG		
*nanA* F	TGGTACAGGTCCTGGTGTTGG	204	49
*nanA* R	ACGCTGATTTGTTCGAAGCGT		
*nanA* probe	TGGGATTTGGGCGCTAGCCCT		
*gapdh* F	GCTGCCATTGCAGGCTTAGG	209	61
*gapdh* R	GGTTGCCACGCGTGTATCTG		
*gapdh* probe	CGCTCAATGACCACGGTCAGCGCA		
*gyrB* F	GGCTGCGGAAAATCCACGTT	152	61
*gyrB* R	CACTTTACCGCAGTCCACGC		
*gyrB* probe	ACTTGCCCCAACTGAACACCACGA		

F and R refer to forward and reverse primers, respectively, bp = base pair.

**Table 2:** General features of the genomic organization of *PMTB2.1*.

Feature of the Sequence of *PMTB2.1*	Description
Genome size	2,315,13
Genome GC content	40.32%
*oriC* length	415 nucleotides
Putative *oriC* location	1,837,028–1,837,442
Total CDs	2097
RNA gene	79
16S rRNAs	6
23S rRNAs	6
5S rRNAs	7
tRNAs	56
ncNAs	4

CDs = DNA coding sequences; *oriC* = origin of replication; rRNA = ribosomal RNA; tRNA = transfer RNA; ncRNA = non-coding RNA.

**Table 3:** Genomic distance of *PMTB2.1* compared to other selected genomes.

Bacteria Strain Name	*PMTB2.1*	*K12* *MG1655*	*PM 70*	*JL03*	*SH0165*	*PM* *36950*	*PM* *HN06*	*PM 3480*	*PM HB01*
*P. multocida* *PMTB2.1*	0.00	0.80	0.08	0.72	0.72	0.10	0.11	0.09	0.12
*Escherichia coli* *K-12 MG1655*	0.80	0.00	0.81	0.80	0.82	0.81	0.81	0.80	0.81
*P. multocida* *PM70*	0.08	0.81	0.00	0.72	0.72	0.09	0.10	0.08	0.12
*A. pleuropneumoniae* *JL03*	0.72	0.80	0.72	0.00	0.68	0.72	0.72	0.72	0.73
*H. parasuis* *SH0165*	0.72	0.82	0.72	0.68	0.00	0.72	0.72	0.72	0.73
*P. multocida* *36950*	0.10	0.81	0.09	0.72	0.72	0.00	0.12	0.10	0.06
*P. multocida* *HN06*	0.11	0.81	0.10	0.72	0.72	0.12	0.00	0.07	0.13
*P. multocida* *3480*	0.09	0.80	0.08	0.72	0.72	0.10	0.07	0.00	0.12
*P. multocida* *HB01*	0.12	0.81	0.12	0.73	0.73	0.06	0.13	0.12	0.00

Note. Genomic distances were calculated for all nine genomes. Pairwise coverage values are subtracted from one to yield a distance value. The value closest to one is more distant from *Pasteurella multocida*.

**Table 4:** Comparison of *P. multocida* strain *PMTB2.1* and complete genome sequences of other *P. multocida* of different serotypes.

*Pasteurella* Species	GenBank Accession No.	Host and Serotype	Genome Size (bp)	GC Content (%)	Reference	Compared with *PMTB2.1*
*P. multocida* strain *PM70*	AE004439.1	Avian (F)	2,257,487	40.4	May et al., 2001	57,651 bp less
*P. multocida* strain *36950*	CP003022.1	Bovine (A)	2,349,518	40.4	Michael et al., 2011	34,380 bp more
*P. multocida* strain *3480*	CP001409.1	Swine (A)	2,378,127	40.3	Unpublished	62,989 bp more
*P. multocida* strain *HN06*	CP003313.1	Swine (D) toxigenic	2,402,218	40.2	Liu et al., 2012	87,080 bp more
*P. multocida* strain *HB01*	CP006976	Bovine (A)	2,416,068	40.3	Peng et al., 2016	100,930 bp more
*P. multocida* strain *PMTB2.1*	CP007205.1	Buffalo (A)	2,315,138	40.4	This study	-

**Table 5:** The tad locus of *PMTB2.1*.

No	Gene ID	Annotation	Gene Name
1	AW43_04840	pilus assembly protein TadG	*tadG*
2	AW43_04845	protein Tad F	*tadF*
3	AW43_04850	protein TadE	*tadE*
4	AW43_04855	NrfG protein	*tadD*
5	AW43_04860	pilus assembly protein TadC	*tadC*
6	AW43_04865	MaoC protein	*tadB*
7	AW43_04870	pilus assembly protein CpaF	*tadA*
8	AW43_04875	pilus assembly protein Protein: (Flp pilus assembly protein, ATPase CpaE)	*tadZ*
9	AW43_04880	protein RcpB	*rcpB*
10	AW43_04885	Secretin	*rcpA*
11	AW43_04890	flp operon protein C	*rcpC*
12	AW43_04895	flp operon protein B	*tadV*
13	AW43_04900	pilus assembly protein	*flp2*
14	AW43_04905	fimbrial protein	*flp1*

Details of *tad* locus genes in *PMTB2.1*, all of the 14 genes with gene ID and annotation result.

**Table 6:** Relative fold change expressions of selected genes of *PMTB2.1* at different time points.

Time Points	Relative Fold Changes of Iron-Related Genes and *nanA* Gene in Iron-Limited Environment				
	*fbpB*	*fecE*	*fur*	*yfeA*	*nanA*
**30**	3.38 ^b^ ± 0.19	281.24 ^b^ ± 1.23	4.80 ^b^ ± 0.24	26.02 ^b^ ± 0.11	1.41 ± 0.31
**60**	25.69 ^b^ ± 1.50	4.70 ^a^ ± 0.36	2.88 ^a^ ± 0.07	12.11 ^a^ ± 0.10	1.09 ± 0.31
**120**	−1.13 ^a^ ± 0.50	−1.39 ^b^ ± 0.32	4.82 ^b^ ± 0.29	42.11 ^b^ ± 0.42	−1.67 ± 0.40
**180**	−4.95 ^b^ ± 0.59	−1.58 ^b^ ± 1.09	5.46 ^a^ ± 0.05	11.65 ^b^ ± 0.12	1.04 ± 1.50

Note. Negative values indicate down-regulation while positive values indicate up-regulation of genes at various time points assessed with the iron-limited environment. Results are presented as fold changes calculated using the 2–ΔΔCt method of treated samples compared to untreated samples at different time points and normalized to the expression of two house-keeping genes. Relative fold changes with a different letter representing significance (≤0.05) among different time points. The results are from three biological replicates and each biological replicate with three technical replicates.

## Supplementary Materials

The following are available online at http://www.mdpi.com/2073-4425/10/2/81/s1, Table S1: List of virulence genes including the *tad* locus, LPS and capsular genes identified based on VFDB database; Table S2: Genes detected in *PMTB2.1* based on CARD; Table S3: PCR efficiency and co-efficient of the variance of real-time PCR assays; Figure S1: Work flow for the identification of virulence-associated genes and antibiotic resistance gene in *P. multocida* strain *PMTB2.1*; Figure S2: Details of prophage sequence in *PMTB2.1*. The complete genome sequence of *P*. *multocida* strain *PMTB2.1* was deposited in the National Center for Biotechnology Information (NCBI) accession number CP007205.2. The complete genome sequence of *PMTB2.1* and eight selected genomes for comparative analysis is available on NCBI and can be accessed using the accession number mentioned in Table 4.
